# Qualifications of a Hospital Secretary

**Published:** 1924-04

**Authors:** 


### QUALIFICATIONS OF A HOSPITAL SECRETARY.

A HOSPITAL secretary should be a man of many parts. His qualifications are partly natural and inherent and partly acquired. The chief natural qualifications are, first of all, a bright and optimistic spirit, a sense of his high calling and a capacity for hard work coupled with a realisation that it will never bring any great pecuniary advantage to himself. Absolute devotion to the hospital he serves and a constant and unremitting watchfulness over its interests characterise the good hospital officer. Particularly is it essential that he should never be satisfied with things as they are. His thoughts should continually be directed towards finding the better way, whether it be to get money, to spend it, or in the more efficient conduct of the internal affairs of his hospital. A knowledge of human kind in all its classes and an ability to inspire others are great assets.

### The Value of Training.

Hospital officers are not unanimously agreed that a long training in hospital work is absolutely essential, but, other things being equal, the man with a varied and comprehensive experience gained in subordinate positions is certainly to be preferred. Unfortunately, in many cases he is not preferred and the whole hospital service thereby loses. There is no encouragement for the junior man. A knowledge of business methods, hospital accountancy, building and structural repairs and some acquaintance with the work of other departments are undeniably useful. Methods and standards vary enormously in different hospitals and it seems reasonable to assume that a training gained at more than one hospital is advantageous. Some hospitals do one thing well. At nearly all, something can be learned.

### The Secretary and His Committee.

A secretary can often be judged by his relations with the committee that governs his hospital. It is primarily his business to gain their confidence. They should respect his judgment. After all, he is a professional. They, in many cases, are immersed in their own business affairs and cannot be so well informed in hospital matters. As far as possible, the secretary should have made up his mind on the questions for discussion before he goes into the committee room. He will not always get his own way, but he will often do so, and if he is wise and tactful the members of his committee will think that they have made the suggestions and that their decisions have been acted upon.

### Opportunities for Propaganda.

A good hospital secretary, particularly in a large institution, will not concern himself too deeply with routine work. He will delegate much of that to others—his work is that of supervision. His duty is to direct the work of his subordinates, to think out the problems of the hospital, to watch its progress and to guide its policy. He should have a keen eye for opportunities. Propaganda should be his strong point. He will be more concerned with the income than the expenditure. The careful control of expenditure is important enough, but the real problem is to make the income meet the outgoings. A hospital cannot cut its coat according to its cloth. He should be able to create wide interest in and many friends for his hospital. If interest and good will are sought and found, money will follow as surely as night follows day. Finally, he should never grow weary or stale or think that all has been done that can be done. The voluntary system gives wide scope and the financial difficulties of many hospitals (especially provincial ones) can be solved by better organisation to secure a really wide basis of support.

